# Effect of long chain omega-3 polyunsaturated fatty acids on inflammation and metabolic markers in hypertensive and/or diabetic obese adults: a randomized controlled trial

**DOI:** 10.3402/fnr.v60.29268

**Published:** 2016-01-29

**Authors:** Mohammed S. Ellulu, Huzwah Khaza'ai, Ismail Patimah, Asmah Rahmat, Yehia Abed

**Affiliations:** 1 Department of Nutrition and Dietetics, Faculty of Medicine and Health Sciences, Universiti Putra Malaysia, Serdang, Selangor, Malaysia; 2 Department of Biomedical Science, Faculty of Medicine and Health Sciences, Universiti Putra Malaysia, Serdang, Selangor, Malaysia; 3 Faculty of Public Health, Al Quds University of Gaza, Gaza City, Palestine

**Keywords:** obesity, hypertension, diabetes, inflammation, omega-3 fatty acids, EPA, DHA

## Abstract

**Background:**

Obesity is a degree of excess weight that predisposes people to metabolic syndromes via an inflammatory mechanism. Hypertensive and diabetic people have higher risks of developing systemic inflammation. Long chain omega-3 polyunsaturated fatty acids (LC ω-3 PUFAs) can reduce the cardiovascular events and help against inflammation.

**Objective:**

To identify the effects of LC ω-3 PUFAs on reducing the levels of inflammatory markers on hypertensive and/or diabetic obese adults.

**Materials and methods:**

Sixty-four patients, who were hypertensive and/or diabetic obese with high levels of inflammatory markers, from primary healthcare centers of Gaza City, Palestine, enrolled in two groups of an open-label, parallel, randomized, controlled trial for 8 weeks. Thirty-three patients were in the control group, and 31 patients were in the experimental group. The experimental group was treated with a daily dose of 300 mg eicosapentaenoic acid and 200 mg of docosahexaenoic acid.

**Results:**

Treatment with LC ω-3 PUFAs significantly reduced the level of high sensitivity C reactive protein (hs-CRP) [14.78±10.7 to 8.49±6.69 mg/L, *p*<0.001], fasting blood glucose (FBG) [178.13±58.54 to 157.32±59.77 mg/dL, *p*=0.024], and triglyceride (TG) [209.23±108.3 to 167.0±79.9 mg/dL, *p*<0.05] after 8 weeks of treatment, whereas no significant changes appeared in interleukin 6 (IL-6) and total cholesterol (TC). In the control group, significant reduction was detected for FBG [187.15±64.8 to 161.91±37.9 mg/dL, *p*<0.05] and TG [202.91±107.0 to 183.45±95.82 mg/dL, *p*<0.05], and no changes for hs-CRP, IL-6, or TC. By comparing the experimental group with the changes of control group at the endpoint, LC ω-3 PUFAs did not reach the clinical significance in treating effectiveness for any of the clinical variables.

**Conclusion:**

LC ω-3 PUFAs have recommended effects on health; the obtained results can improve the role of LC ω-3 PUFAs as a protective factor on inflammation and metabolic dysregulation. The time allowed or the dose used could be insufficient to achieve full treatment affectivity.

In the Eastern Mediterranean region, the state of obesity has reached an alarming level (1). According to the *First National Health and Nutrition Survey* (1999–2000) in Palestine, the overall prevalence of overweight was 62.4%, and 24.4% of whom are obese (2). Specifically in the Gaza Strip, 57.0, 66.8, and 67.5% indicated the prevalence of being overweight including obese in urban areas, refugee camps, and rural areas, respectively (3).

Obesity is a risk factor for developing different diseases including metabolic diseases (4) and diabetes (5). The hypothesized physiological mechanism is the state of chronic low-grade inflammation that is associated with excess adipose tissue (6). The link between obesity and inflammation has been derived from the finding that proinflammatory cytokines tumor necrosis factor alpha (TNF-α) and interleukin 6 (IL-6) are overexpressed in obesity (7). The high levels of cytokines generally and IL-6 particularly are associated with decreased nitric oxide and increased reactive oxygen species, which lead to endothelial and microvascular dysfunction (8). Accordingly, increased serum level of IL-6 stimulates the liver to synthesize and secrete the systemic inflammatory marker C reactive protein (CRP) (9).

On the other hand, the antioxidant defense factors become lower due to accumulation of fat in adipose tissues and the associated production of reactive oxygen species (10). Because of that, nutritional intervention has been recognized as a possible method to restore the antioxidant levels and to treat the inflammatory status and metabolic dysregulation in diabetic or cardiovascular disease (CVD) patients (11, 12). Micronutrients such as vitamins and trace elements are required at appropriate intakes for the immune system to function optimally (13).

Marine foods consumption is associated with reduced CVD risk (14), cerebrovascular disease (15), cancer (16), depression (17), and inflammation (18). The effect of fish is believed to be mainly due to its component of long chain omega-3 polyunsaturated fatty acids (LC ω-3 PUFAs) (11). They are represented in the most biologically important forms; docosahexaenoic acid (DHA) and eicosapentaenoic acid (EPA) (19). EPA/DHA supplement has the potency to treat many diseases. LC ω-3 PUFAs have several important biological effects on a range of cellular functions that may reduce the onset of heart diseases and reduce mortality among patients with coronary heart disease, possibly by stabilizing the heart's rhythm and by reducing blood clots (20). CVD, diabetes, and metabolic dysregulation have been linked strongly to inflammation (8).

Therefore, the objective of the study reported here is to evaluate the effect of LC ω-3 PUFAs on inflammatory markers (serum level of IL-6 and CRP) in hypertensive and/or diabetic obese adults. We also aimed to find out the change in metabolic markers; fasting blood glucose (FBG) and lipid profile factors, total cholesterol (TC), and triglyceride (TG) after 8 weeks of LC ω-3 PUFAs intervention.

## Subjects and methods

### Study population

Eligible participants for this study were obese [body mass index, BMI (kg/m^2^) ≥30], hypertensive, and/or diabetic patients between 28 and 60 years of age who systemically visited the primary healthcare centers to follow up at three locations in Gaza City, Palestine. The diagnosis and selection of hypertensive and diabetic cases depended on patients’ self-reporting and proof of the patients’ medical history records obtained from the healthcare center. For instance, all of the diabetic patients were type 2 diabetes mellitus (T2DM). Participants were eligible if they had a high CRP level (high-sensitivity CRP [hs-CRP] ≥6 mg/L; *in titer test, CRP <6 mg/L appeared as negative*). Patients were excluded if they had any acute illness within the past 2 weeks, regularly used non-steroidal anti-inflammatory drugs (naproxen and cyclooxygenase-2 inhibitors) and/or cholesterol-lowering agents (statins), suffered from systemic and inflammatory diseases that change physical or laboratory tests (arthritis, renal, thyroid, hepatic, respiratory, gout, active malignant disease, pregnancy, breastfeeding), and/or documented intolerability to LC ω-3 PUFAs.

The recruitment of study participants began in November 2013 and was completed in May 2014. Of 484 screened patients, 108 were enrolled in the study and assigned randomly to one of the three groups: control or one of two experimental/intervention groups – LC ω-3 PUFAs treatment and vitamin C treatment.

### Study design and intervention

The clinical trial was randomized, open-labelled, controlled, and parallel, and performed in compliance with the World Medical Association (WMA) Declaration of Helsinki: Ethical Principles for Medical Research Involving Human Subjects adopted by the 18th WMA General Assembly, Helsinki, Finland, June 1964, and amended by 59th WMA General Assembly, Seoul, Korea, October 2008 (number: PHRC/HC/11/13, March 4, 2013). The trial was registered and approved by Universiti Putra Malaysia [Ref. No.: FPSK_Mac (13)04 (21 June 2013)]. It was conducted at the Ministry of Health primary health care centers in Gaza City, Palestine, and followed the Good Clinical Practice guidelines of the Department of Clinical Research, Faculty of Public Health, Al-Quds University.

Recruited patients were randomly assigned to LC ω-3 PUFAs treatment (experimental) group or control group using manual blocks formation based on the rolling of a dice. The allocation and randomization of randomized controlled trial (RCT) patients depended on a blocking system; 108 patients diseased with hypertension (HT) and/or T2DM were selected and arranged in ascending order. Then, restricted randomization for three groups (named as A, referred to vitamin C group; B, referred to LC ω-3 PUFAs group; and C, referred to control group); the created blocks of sequences ensured equal numbers of allocated participants. For three groups, six blocks created as the following: 1) ABC, 2) ACB, 3) BAC, 4) BCA, 5) CAB, and 6) CBA. After that, rolling of dice got number 1; means block number 1, which is ABC, so, the first participant was allocated in group A, the second in group B, and the third in group C, and finally repeated rolling of dice took place to allocate all of RCT patients in the groups.

Patients in the experimental group of current study were assigned to treatment with 1.0 g fish oil from sardine and anchovy per day, whereas the control group was kept free from supplements. The fish oil containing LC ω-3 PUFAs were supplied in softgel capsules (commercial formula) including 300 mg EPA and 200 mg DHA. The daily dose of fish oil was higher than Samimi et al. (21) who examined the effect of 1.0 g fish oil involving 180 mg EPA and 120 mg DHA per day for 6 weeks on hs-CRP in gestational DM through placebo RCT. We used a higher dose of EPA/DHA to achieve higher variance and to keep pace with longer intervention period.

Participants in both groups were advised to maintain constant daily lifestyle habits unless they progressed to any of the excluding criteria. Based on an adapted form of Global Physical Activity Questionnaire (v 2) (22), the majority of participants indicated that they undertook a low level of physical activity (PA), whereas the remaining participants indicated they undertook a moderate level of PA. None of the participants said they undertook a high level of PA before or during the trial. The participants were approximately equally distributed in terms of smoking habit, which was evaluated by an adapted and modified form of the Behavioral Risk Factor Surveillance System (23). In terms of smoking status, each patient was categorized either as a smoker, ex-smoker, passive smoker, or non-smoker.

The intervention continued for 8 weeks, and the participants returned for follow-up visits every 2 weeks. At study baseline (randomization time), we performed anthropometric measurements (BMI, waist circumference [WC], as well as blood pressure [systolic and diastolic]) and blood sampling. We measured systemic inflammatory markers (hs-CRP and IL-6) and metabolic markers (FBG, TC, and TG).

Sample size was estimated as 32 per a group to detect a reduction in inflammatory and metabolic markers at a *p*-value 0.05 with a power of 90%, and a dropout rate of 10% was expected. Thus, we aimed to recruit 72 patients.

### Tools

A Seca stadiometer was used to assess height and weight to obtain BMI according to the World Health Organization classification (24) (underweight: <18.5 kg/m^2^; normal weight: ≤25.0 kg/m^2^; overweight: ≤30.0 kg/m^2^; obese: ≥30 kg/m^2^), and a Seca 201 non-elastic tape was used to determine WC according to the National Institute for Health and Clinical Excellence classification (25) (male: normal <102 cm, high ≥102 cm; female: normal <88 cm, high ≥88 cm).

Quantitative method was used to assess FBG, TC, TG, and hs-CRP. A CRP turbidimetric latex 1:5 kit was used to assess hs-CRP, an enzymatic colorimetric method with glucose oxidase was used to estimate FBG, and commercial kits were used to assess TC and TG. All quantitative data were evaluated using a Mindray BS-120 Chemistry Analyzer. IL-6 was assessed using enzyme-linked immunosorbent assay kits (Sigma–Aldrich) via one-run reading.

Blood was collected by a trained nurse or physician of the health center. A 7-mL blood sample was drawn into a polyethylene evacuated tube and then divided into two separate tubes. One of the tubes of blood was used to evaluate quantitative biochemical measures (hs-CRP, FBG, TC, and TG), and the other was stored at −80°C, after separation of serum, for analysis through one-run of an enzyme-linked immunosorbent assay reader to assess IL-6.

All participants completed an informed consent form before attending the study sessions and blood sample collection. All participants were assured of confidentiality and any required information was provided to them. It was clearly explained to them that participation would be voluntary and they could withdraw their participation at any time. A case report form for each patient was used for data handling and record keeping.

Every attempt was made to minimize biases and to conduct the study in the most ethical manner possible.

### Withdrawal and monitoring criteria

We established withdrawal criteria to manage the response and dropout of participants. Patient follow-ups were discontinued if the participant withdrew consent or became non-cooperative; the participant developed an illness or condition that changed physical or laboratory tests, as explained earlier in the exclusion criteria; the study supplements resulted in the development of intolerable adverse effects; the participant vomited the supplement within 4 h of administration; and/or a female participant became pregnant.

Additionally, the monitoring criteria were used to evaluate patients’ involvement and compliance. The supplement pills were distributed to the participants every 2 weeks, participants were required to keep a diary, their entries were monitored, and participants were advised to make a daily notice in their own calendar or cell phone to remind them of the dose time. The researcher also conducted a random contact with a number of participated patients in both groups, at the time the patients advised, to inform the researcher about any progression.

### Statistical analysis

Data were analyzed using SPSS software (v 21.0; IBM Corporation, Armonk, NY, USA). Descriptive statistics, including χ^2^, were used to compare the categorical variables of subjects’ characteristics in both groups. After the assumption of normality, continuous variables were presented as mean±standard deviation. Independent *t-*tests were used to assess the difference between the groups at the baseline of randomization and endpoint of study. Paired *t*-tests were used to evaluate the difference within the groups. A *p*-value of ≤0.05 was considered statistically significant and the level of confidence was 95% at a power equal to 90%.

## Results

### Study population and intervention

**Fig. 1:**
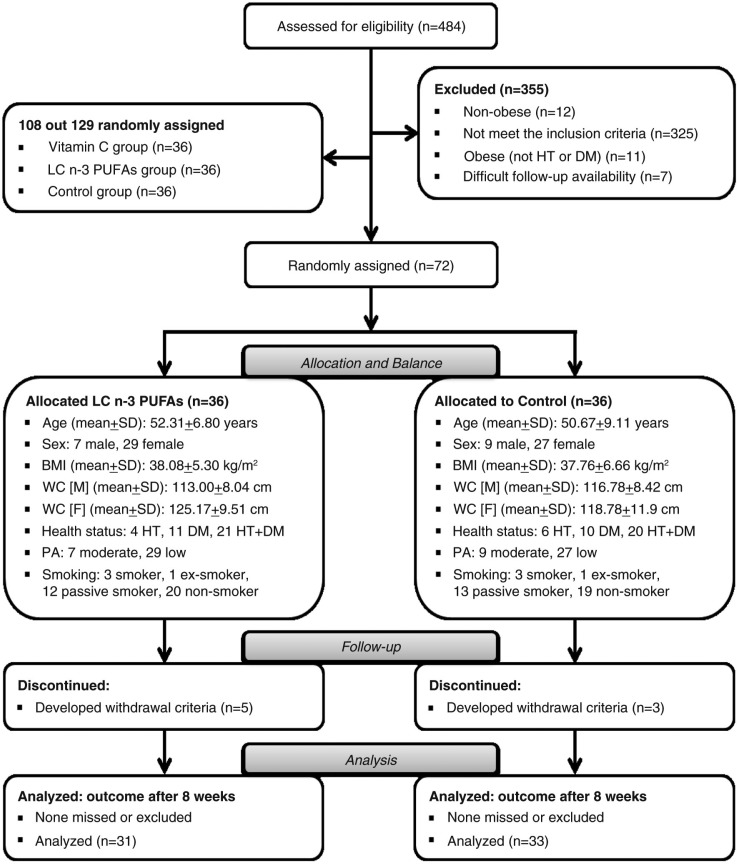
Consolidated Standards of Reporting Trials (CONSORT) flowchart. BMI, body mass index; DM, diabetes mellitus; HT, hypertension; LC ω-3 PUFAs, long-chain omega-3 polyunsaturated fatty acids; PA, level of physical activity; SD, standard deviation; WC-F, waist circumference – females; WC-M, waist circumference – males.

The trial aimed to evaluate the effect of LC ω-3 PUFAs on inflammatory (hs-CRP and IL-6) and metabolic markers (FBG, TC, and TG) in hypertensive and/or diabetic obese patients. This paper reports on two groups, the control and LC ω-3 PUFAs treatment groups. The effect of vitamin C treatment was discussed in Ellulu et al. (26). The progression of patients through the study is shown in Fig. 1. Sixty-four patients completed the study (31 patients in the intervention group and 33 in the control group). Eight patients were dropped during follow up because they met one or more of the withdrawal criteria (5 patients in the intervention group out of 36 patients and 3 in the control group out of 36 patients). The drop out from the analysis was attributed to poor cooperation, and to the development of illness or a condition might change the laboratory tests such as infection and using excluded medicines.

### Balance at baseline

The baseline demographic (age, sex, smoking, level of PA, and health status/disease), anthropometric (BMI and WC), and clinical characteristics (hs-CRP, IL-6, FBG, TC, and TG) of the patients in both groups were comparable (Table 1). In total, 72 patients were recruited at the baseline to participate in the study. To assess the balance between the groups, comparisons of all patients’ characteristics were undertaken.

**Table 1:** Demographic, anthropometric, and clinical variables at baseline (randomization) in both groups

	LC ω-3 PUFAs group (*n*=36)	Control group (*n*=36)	Test value	*p*
Characteristics and lifestyle				
Age, years (mean±SD)	52.31±6.80	50.67±9.11	*t*=0.864^a^	0.391
Sex (male, female)	[7, 29]	[9, 27]	χ^2^=0.321^b^	0.778
Smoking (S, ES, PS, NS)	[3, 1, 12, 20]	[3, 1, 13, 19]	χ^2^=0.066^b^	0.996
PA (moderate, low)	[7, 29]	[9, 27]	χ^2^=0.321^b^	0.778
Disease (HT, DM, HT+DM)	[4, 11, 21]	[6, 10, 20]	χ^2^=0.472^b^	0.790
Anthropometric characteristic				
BMI, kg/m^2^ (mean±SD)	38.08±5.30	37.76±6.66	*t*=0.221^a^	0.826
WC-M, cm (mean±SD)	113.00±8.04	116.78±8.42	*t*=–0.907^a^	0.380
WC-F, cm (mean±SD)	125.17±9.51	118.78±11.9	***t***=**2.220^a^**	0.031*
WC, overall (mean±SD)	122.81±10.3	118.28±11.1	*t*=1.788^a^	0.078
Clinical characteristics				
hs-CRP, mg/L (mean±SD)	14.04±10.1	14.02±13.7	*t*=0.008^a^	0.994
IL-6, pg/mL (mean±SD)	1.79±0.66	1.97±0.72	*t*=–1.063^a^	0.292
FBG, mg/dL (mean±SD)	179.56±57.18	187.83±65.92	*t*=–0.569^a^	0.751
TC, mg/dL (mean±SD)	195.92±31.90	208.72±38.27	*t*=–1.542^a^	0.128
TG, mg/dL (mean±SD)	206.50±104.4	200.42±102.7	*t*=0.249^a^	0.804

S, smoker; ES, ex-smoker; PS, passive smoker; NS, non-smoker; BMI, body mass index; DM, diabetes mellitus; FBG, fasting blood glucose; hs-CRP, high-sensitivity C-reactive protein; HT, hypertension; IL-6, interleukin 6; PA, level of physical activity; SD, standard deviation; TC, total cholesterol; TG, triglyceride; WC-F, waist circumference – females; WC-M, waist circumference – males.

Normal ranges of clinical characteristics according to the American Heart Association and Center for Disease Control and Prevention (AHA/CDC): hs-CRP <3.0 mg/L, IL-6 <1.0 pg/mL, and according to National Cholesterol Education Program and Adult Treatment Panel-III: FBG<110 mg/dL, TC<200 mg/dL, TG<150 mg/dL.

a Independent-samples *t*-test.

b Chi-square test.

No statistically significant difference for any variable of comparison was detected; no difference appeared between the groups in terms of age, sex, smoking habit, level of PA, or health status/disease. In addition, equality between the groups was assumed in terms of the anthropometric measurement of BMI and overall WC. Furthermore, no significant differences were found when comparing the groups in terms of the clinical variables hs-CRP, IL-6, FBG, TC, and TG. These results for all variables indicate balance at baseline between the groups and indicate equality, because the differences did not reach the significance level of a *p*-value ≤0.05.

### Changes in clinical characteristics after 8 weeks

Table 2 presents the changes in patients’ clinical characteristics after 8 weeks, between the end point (after treatment) and baseline (before treatment), for both groups. In LC ω-3 PUFAs group, the changes appear in three variables after 8 weeks of intervention: hs-CRP, FBG, and TG can be seen to have reduced significantly as the following: 14.78±10.7 to 8.49±6.69 mg/L, *p*<0.001; 178.13±58.54 to 157.32±59.77 mg/dL, *p*=0.024; and 209.23±108.3 to 167.0±79.98 mg/dL, *p*=0.002; respectively. In the control group, the change appears in two variables after 8 weeks without added supplements FBG (187.15±64.89 to 161.91±37.97 mg/dL, *p*=0.001) and TG (202.91±107.0 to 183.45±95.82, *p*=0.026). Due to the open-label nature of the study, the changes within the control group could be attributed to changes in lifestyle factors, such as eating habits or level of PA.

**Table 2:** The change in clinical characteristics after 8 weeks of intervention in both groups

	LC ω-3 PUFAs group (*n*=31) (mean±standard deviation)		Control (*n*=33) (mean±standard deviation)		
			
	Before	After	Before	After	*p*
hs-CRP (mg/L)	14.78±10.7	8.49±6.69*	14.50±14.26	11.81±7.33	0.064
IL-6 (pg/mL)	1.73±0.62	1.66±0.88	1.95±0.75	2.01±0.87	0.116
FBG (mg/dL)	178.13±58.54	157.32±59.77*	187.15±64.89	161.91±37.97*	0.714
TC (mg/dL)	196.74±31.89	196.71±26.38	211.03±39.04	213.38±38.77	0.05**
TG (mg/dL)	209.23±108.3	167.00±79.98*	202.91±107.0	183.45±95.82*	0.460

FBG, fasting blood glucose; hs-CRP, high-sensitivity C-reactive protein; IL-6, interleukin 6; SD, standard deviation; TC, total cholesterol; TG, triglyceride.

* Change was significant at the 0.05 level (two-tailed) (calculated by paired samples *t*-test).

** Difference was significant at the 0.05 level (two-tailed) (calculated by independent-samples *t*-test).

At the end of the study, after 8 weeks of treatment, in the LC ω-3 PUFAs group, 31 patients completed the study and 5 were lost to follow up. In the control group, 33 patients completed the study and 3 were lost to follow up. The overall response rate was 88.88%; 86.11% in the LC ω-3 PUFAs group and 91.66% in the control group.

However, the statistically significant changes found within the treatment group before and after the treatment period did not necessarily mean the supplement was effective in reducing the risk of high levels of patients’ clinical characteristics. To determine this, we compared the differences between the groups at the end point (after treatment) to determine the clinical significance of LC ω-3 PUFAs. The results revealed that *p*-value did not reach the significance level for hs-CRP, IL-6, FBG, and TG. That means, the reduction in clinical variables due to the use of LC ω-3 PUFAs did not reach the effective level after 8 weeks of daily intake when the results were compared to the control group. In the case of TC, there is no difference within the group of LC ω-3 PUFAs as shown as in the control group, but the significance achieved in comparing between the groups at the end point (*p*-value ≤0.05) was obtained because the level of TC was raised in the control group and stabilized in the group of LC ω-3 PUFAs after 8 weeks.

## Discussion

Inflammation due to adiposity is the basis of obesity-related systemic inflammation; it predisposes patients to the development of metabolic abnormalities and CVD (8). This RCT intended to find out the effect of LC ω-3 PUFAs on the inflammatory and metabolic markers conducted in Gaza City, Palestine, and the importance of this study rose because it included hypertensive and/or diabetic obese patient. The mechanism of action of LC ω-3 PUFAs as anti-inflammatory has been explained by Adkins and Kelley (27). They respond to inflammation in CVD and atherosclerosis through direct and indirect mechanisms. A direct mechanism through which LC ω-3 PUFAs decrease inflammation includes its rapid effect on the regulation of transcription factors (28), and indirect modes of actions include the production 3- and 5-series eicosanoids (29), inflammation-resolving lipid mediators (30), and suppression of acute phase reactants (9). For more explanation, LC ω-3 PUFAs can inhibit inflammation by reducing TNF-α, IL-6, and CRP, and increasing three- and five-series eicosanoids, lipoxins, resolvins, and protectins, which are derived from LC ω-3 PUFAs (27).

### Effect on hs-CRP

We examined the effect of LC ω-3 PUFAs on inflammatory markers hs-CRP after 8 weeks of daily intake. In the experimental group, hs-CRP reduced significantly, whereas the reduction in the control group did not reach the significance level. Similar evidences are available to support our results. Samimi et al. (21) recruited gestational DM women in placebo RCT, after the supplementation of 180 mg EPA/120 mg DHA for 6 weeks, they obtained a significant difference in hs-CRP between the experimental and placebo groups (–236.3±1541.9 vs. 898.6±2292.7 ng/mL, *p*=0.03). Similarly, Vasil'ev et al. (31) studied the effect of PUFAs in metabolic syndrome manifestation among arterial hypertensive patients, where 22 patients out of 32 received 1.5 g/day of PUFAs for 1 month. The study resulted in a significant decrease of serum hs-CRP (about 40.7% reduction).

In a cross-sectional design, Micallef et al. (32) included 124 free-living adults, dividing them into tertiles of plasma hs-CRP (<1.0, 1.0–3.0 and ≥3.0 mg/L). Plasma hs-CRP concentration was negatively correlated with total LC ω-3 PUFAs; the highest hs-CRP tertile (≥3.0 mg/L) had significantly lower concentrations of total LC ω-3 PUFAs (*p*=0.05).

On the contrary, an insignificant change in hs-CRP was observed in the experimental groups. For instance, Yusof et al. (33) conducted a placebo RCT for 100 patients of carotid endarterectomy; experimental group (47 patients) received 888 mg EPA and 777 mg DHA daily, whereas control group (53 patients) received 2 g of olive oil capsule daily for 21 days. The study obtained no significant change in hs-CRP due to intervention of EPA/DHA in the experimental group.

Similarly, Itariu et al. (34) reported that hs-CRP did not change in the experimental group. The study recruited 55 adults at open-label RCT; they are severely obese non-diabetic patients who have elective bariatric surgery. Twenty-seven of them were selected to have 3.36 g/day of PUFAs for 8 weeks before the surgery and 28 were selected to be controlled with equivalent butterfat intake.

Furthermore, Mackay et al. (35) tested the effect of 850 mg EPA and 882 mg DHA for 6 weeks in reducing the increased platelet and endothelial activity in patients with intermittent claudication. Placebo RCT, which was conducted in 150 patients (73 in experimental group and 77 in control group), concluded that hs-CRP did not change in both experimental and control groups.

To test the efficacy of LC ω-3 PUFAs, the repeated test between experimental and control groups was performed at endpoint; the result from this study revealed insignificant difference of hs-CRP between the groups. Similar evidences are available to support the present result. Flock et al. (36) examined the effect of EPA/DHA on inflammatory markers in 116 healthy adults with low fish intake through RCT; there were no significant effects of supplemented EPA/DHA on hs-CRP after 5 months for the following doses: 0, 300, 600, 900, or 1,800 mg/day EPA/DHA. Similarly, Shaikh et al. (37) approved our results on hs-CRP; the study included two cohorts of CVD patients, the first cohort included patients with normal TG level (90–199 mg/dL) and the second included patients with hypertriglyceridemia (200–500 mg/dL). Each cohort involved experimental and placebo groups in a RCT continued for 8 weeks, the experimental groups received LC ω-3 PUFA formulation of <90% purity with a 6:1 EPA and DHA ratio (4 g/day), and placebo groups received sufficient corn oil. The study resulted in no statistical difference in hs-CRP between experimental and control groups for the both cohorts after 8 weeks of intervention LC ω-3 PUFAs.

Moreover, Skulas-Ray et al. (38) concluded insignificant change on hs-CRP in comparison to the effects of a nutritional dose of EPA+DHA (0.85 g/d) with those of a pharmaceutical dose (3.4 g/day) in 23 men and three post-menopausal women with moderate hypertriglyceridemia (150–500 mg/dL), by placebo-controlled, double-blind, randomized, three-period crossover trial for 8 weeks of treatment.

On the other hand, different evidence is explained by Samimi et al. (21), who examined the effect of 180 mg EPA and 120 mg DHA per day after 6 weeks on hs-CRP in gestational DM through placebo RCT. The compared difference between the experimental group and placebo group after the study resulted in a significant difference and reduction in hs-CRP.

### Effect on IL-6

We examined the effect of LC ω-3 PUFAs on the proinflammatory marker IL-6 after 8 weeks of daily intake. In both experimental and control groups, levels of IL-6 did not change significantly. Similar evidences are available to support the present results.

Darghosian et al. (39) designed a prospective, double-blind, placebo-controlled, parallel group study to study the effect of LC ω-3 PUFAs on inflammatory and oxidative markers involved IL-6 among patients of recurrent atrial fibrillation (AF). Patients with paroxysmal or persistent AF randomized into two groups; 126 were in experimental group (4 g/day PUFAs) and 64 were in control group (placebo). LC ω-3 PUFAs did not result in clinically meaningful changes in concentrations of inflammatory markers. AF recurred in 58.7% of experimental group and 46.9% of control group.

Similarly, the study by Yusof et al. (33) did not result in effective reduction by supplementing 888 mg EPA and 777 mg DHA daily for 21 days on patients of carotid endarterectomy. As well, Mackay et al. (35) did not detect reduction of IL-6 in experimental group (850 mg EPA and 882 mg DHA for 6 weeks) in patients with intermittent claudication. Moreover, Skulas-Ray et al. (38) did not find change after 8 weeks of treatment in IL-6 by comparing the group of nutritional dose (EPA+DHA: 0.85 g/day) with the group of pharmaceutical dose (3.4 g/day).

In contrast, Tousoulis et al. (40) evaluated the effect of 2 g per day of LC ω-3 PUFAs on inflammation and vascular function in subjects with metabolic syndrome for 12 weeks, by cross-over placebo, double-blind RCT, IL-6 reduced significantly in the experimental group while no change resulted in the placebo group.

In addition, Itariu et al. (34) found that the 3.36 g/day of PUFAs for 8 weeks before the bariatric surgery of severely obese can decrease the gene expression of most analyzed inflammatory genes in subcutaneous adipose tissue, increase the production of anti-inflammatory eicosanoids in visceral subcutaneous adipose tissue, and the levels of IL-6 decreased significantly in the experimental group while no change appeared in the control group.

Similarly, Zhao et al. (41) reported that proinflammatory cytokines including IL-6 decreased significantly in a prospective, single-blind, randomized, placebo controlled group including 76 patients of heart failure assigned to two groups; 38 as experimental group (2 g/day: EPA=0.18 g, and DHA=0.12 g), and 38 as placebo group for 3 months.

To test the efficacy of LC ω-3 PUFAs, the repeated test between experimental and control groups was performed at endpoint. The result from this study revealed insignificant difference of IL-6 between the groups. Flock et al. (36) results are consistent with our results; they detected insignificant difference due to the examined EPA/DHA on IL-6 in 116 healthy adults with low fish intake through RCT after 5 months.

### Effect on FBG, TC, and TG

We examined the effect of LC ω-3 PUFAs on metabolic markers FBG, TC, and TG after 8 weeks of daily intake. Levels of FBG and TG reduced significantly in both experimental and control groups. In contrast, the level of TC was not affected by intervention, while no change was detected in the control group. To view clinical significance, repeated tests between experimental and control groups to identify the efficacy of LC ω-3 PUFAs revealed insignificant change between the groups for FBG and TG, while significance was viewed in test of TC. In the following studies, the role of LC ω-3 PUFAs discussed the effect on the metabolic markers FBG, TC, and TG by clinical RCTs.

Shaikh et al. (37) tested the effect of 4 g/day of PUFAs in experimental groups compared to corn oil as placebo for control groups in the two cohorts (normal TG and high TG) continued for 8 weeks. In both cohorts, statistical and clinical significance detected for the change of TG, this result is absent for FBG and TC. Furthermore, Samimi et al. (21) examined the effect of 180 mg EPA and 120 mg DHA per day for 6 weeks, in lipid profile including TC and TG on gestational DM through placebo RCT; no significant change has been achieved between the groups for FBG, TC, or TG.

Additionally, Tousoulis et al. (40) tested the effect of PUFAs for 12 weeks in lipid profile markers TC and TG, and FBG among metabolic syndrome patients for 12 weeks through RCT. All metabolic markers reduced significantly in the experimental group, while no significant reduction was detected in the placebo group. Furthermore, Yusof et al. (33) proved a positive effect of PUFAs on lipid profile markers in RCT of carotid endarterectomy patients.

Moreover, Itariu et al. (34) assessed the change on metabolic markers; FBG and TC did not show variation after the intervention for both groups, at the time TG got significant variation in control group only. Similarly, Skulas-Ray et al. (38) tested the effect of high dose of PUFAs in FBG and lipid profile; TG reduced significantly with intervention for 8 weeks, while FBG and TC did not affect the supplementation.

Maki et al. (42) recruited 31 men and women to investigate omega-3-acid ethyl esters (4 g/day) on lipid profile through double-blind, randomized cross-over study. TG reduced significantly by 18.8% after 6 weeks, while TC did not achieve a significant change. Finally, Vasil'ev et al. (31) achieved significant reduction in TG (37.2%) due to supplementation of 1.5 g/day PUFAs in arterial hypertensive patients.

## Conclusion

Before starting the intervention of LC ω-3 PUFAs, data showed the balance between the groups to be approximately equal through patients’ characteristics and clinical variables. Minor subgroup inequality was detected for female WC when the vast major of balances have been achieved. Few studies have used repeated tests to evaluate clinical significance like Samimi et al. (21) and Shaikh et al. (37). The disparities in the discussed results supported our results to be consistent with a part of results of the previous studies and inconsistent with the other parts for both inflammatory and metabolic markers. LC ω-3 PUFAs changed the level of hs-CRP, FBG, and TG significantly. However, these reductions are insufficient to consider the LC ω-3 PUFAs as a treating factor of inflammation or metabolic dysregulation. The clinical significance of TC was achieved because the intervention factor stabilized the level of TC in the experimental group at the time the control group got an increase after 8 weeks. Our results evidenced by TC significance suggested the role of LC ω-3 PUFAs as a protective and prophylaxis factor in hypertensive and diabetic obese patients. The concluded affectivity of LC ω-3 PUFAs may be attributed to the short time of study or the dose used. Further studies are needed with variation on the duration and dosage to obtain more explanation on the role of EPA and DHA in inflammatory and metabolic markers.
